# Supplementary material for: Ecological Inference From Isolated Vertebrae: Evaluating Functional Signal Across the Carnivoran Spine
Source: J Morphol. 2025 Dec 30;287(1):e70109. doi: 10.1002/jmor.70109 (PMC12750969; doi:10.1002/jmor.70109)
Supplement: Supplementary file 3 — Vertebrae_locomotion_supplements. [file JMOR-287-e70109-s001.pdf]

Supplementary information for:

Ecological inference from isolated vertebrae: Evaluating functional signal across the carnivoran spine

Julia A. Schwab [1,2], Borja Figueirido[3], Katrina E. Jones[2,4]

[1] Oxford University Museum of Natural History, University of Oxford, Parks Rd, OX1 3PW Oxford, United Kingdom

[2] Department of Earth and Environmental Sciences, University of Manchester, M13 9PL Manchester, United Kingdom

[3] Departamento de Ecología y Geología, Facultad de Ciencias, Campus de Teatinos s/n, Universidad de Málaga, 29071-Málaga, Spain

[4] School of Earth Sciences, University of Bristol, Bristol BS8 1RJ, UK.

1. Specimen List

Suppl. Table 1. Detailed information on the scanned specimens; their museum specimen numbers, source and assigned hunting behaviour/running speeds.

Taxa	Family	ID	Source	Hunting behaviour	Running speed
Ailurus fulgens	**Ailuridae**	UVA 1802	Figueirido et al. 2021	Occasional	slow
Canis lupus	**Canidae**	UVA 4589	Figueirido et al. 2021	Pursuit	fast
Chrysocyon brachyurus	**Canidae**	NMS Z.2015.177	Figueirido et al. 2021	Pounce	fast
Cuon alpinus	**Canidae**	UVA 7106	Figueirido et al. 2021	Pursuit	fast
Lycaon pictus	**Canidae**	UVA 1167	Figueirido et al. 2021	Pursuit	fast
Speothos venaticus	**Canidae**	NMS Z.2015.96.4	Figueirido et al. 2021	Pounce	intermediate
Vulpes vulpes	**Canidae**	UVA 150	Figueirido et al. 2021	Pounce	fast
Otocyon megalotis	**Canidae**	NMS PH107.04	Scanned by JAS at the National Museum of Scotland, Edinburgh	Pounce	intermediate
Vulpes lagopus	**Canidae**	NMS A.F.I.	Scanned by JAS at the National Museum of Scotland, Edinburgh	Pounce	intermediate
Canis lupus	**Canidae**	MU 073	Scanned by Logan Anders, University of Manchester	Pursuit	fast
Canis lupus	**Canidae**	MU 071	Scanned by Logan Anders, University of Manchester	Pursuit	fast
Vulpes vulpes	**Canidae**	MU 091a	Scanned by Logan Anders, University of Manchester	Pounce	fast
Cryptoprocta ferox	**Eupleridae**	NMS EA36.08	Figueirido et al. 2021	Pounce	fast
Acinonyx jubatus	**Felidae**	UVA, NN	Figueirido et al. 2021	Pursuit	fast
Caracal caracal	**Felidae**	UVA 1556	Figueirido et al. 2021	Ambush	fast
Felis silvestris	**Felidae**	UVA NN	Figueirido et al. 2021	Ambush	fast
Leptailurus serval	**Felidae**	UVA 6070	Figueirido et al. 2021	Ambush	fast
Lynx canadiensis	**Felidae**	UVA Z.2001.117.9	Figueirido et al. 2021	Ambush	fast
Neofelis nebulosa	**Felidae**	NMS Z.2001.8	Figueirido et al. 2021	Ambush	fast
Panthera leo	**Felidae**	UVA NN	Figueirido et al. 2021	Ambush	fast
Panthera pardus	**Felidae**	UVA 1600	Figueirido et al. 2021	Ambush	fast

Panthera tigris	**Felidae**	MNCN 21574	Figueirido et al. 2021	Ambush	fast
Panthera tigris altaica	**Felidae**		Figueirido et al. 2021	Ambush	fast
Puma concolor	**Felidae**	UVA 409	Figueirido et al. 2021	Ambush	fast
Lynx rufus	**Felidae**	NMS R6800	Scanned by JAS at the National Museum of Scotland, Edinburgh	Ambush	interm ediate
Panthera onca	**Felidae**	NMS Z.2013.162	Scanned by JAS at the National Museum of Scotland, Edinburgh	Ambush	fast
Panthera uncia	**Felidae**	NMS Z.2009.024.001	Scanned by JAS at the National Museum of Scotland, Edinburgh	Ambush	fast
Panthera leo	**Felidae**	MU 127	Scanned by Logan Anders, University of Manchester	Ambush	fast
Cynictis penicillata	**Herpestidae**	UVA 6730	Figueirido et al. 2021	Pounce	slow
Herpestes ichneumon	**Herpestidae**	UVA 7344	Figueirido et al. 2021	Pounce	slow
Suricatta suricatta	**Herpestidae**	UVA 3098	Figueirido et al. 2021	Pounce	interm ediate
Suricatta suricatta	**Herpestidae**	UVA 7103	Figueirido et al. 2021	Pounce	interm ediate
Crocuta crocuta	**Hyaenidae**	UVA 4215	Figueirido et al. 2021	Pursuit	fast
Hyaena hyaena	**Hyaenidae**	UVA 2981	Figueirido et al. 2021	Pounce	interm ediate
Proteles cristatus	**Hyaenidae**	NMS PH37.98	Figueirido et al. 2021	Occasion al	interm ediate
Martes foina	**Mustelidae**	UVA 7342	Figueirido et al. 2021	Pounce	interm ediate
Mustela putorius	**Mustelidae**	UVA 7203	Figueirido et al. 2021	Pounce	interm ediate
Mustela putorius	**Mustelidae**	UVA 7343	Figueirido et al. 2021	Pounce	interm ediate
Martes foina	**Mustelidae**	NMS R21399	Scanned by JAS at the National Museum of Scotland, Edinburgh	Pounce	interm ediate
Meles meles	**Mustelidae**	MU 056	Scanned by Logan Anders, University of Manchester	Occasion al	slow
Meles meles	**Mustelidae**	MU 125a	Scanned by Logan Anders, University of Manchester	Occasion al	slow
Nasua narica	**Procyonidae**	UVA 5542	Figueirido et al. 2021	Occasion al	slow
Potos flavus	**Procyonidae**	UVA 124	Figueirido et al. 2021	Occasion al	slow

Procyon lotor	**Procyonidae**	UVA 4264	Figueirido et al. 2021	Occasion al	slow
Nasua nasua	**Procyonidae**	NMSZ.2015.163.2	Scanned by JAS at the National Museum of Scotland, Edinburgh	Pounce	interm ediate
Ailuropoda melanoleuca	**Ursidae**	NMS Z.1986.19	Figueirido et al. 2021	Occasion al	slow
Ursus arctos	**Ursidae**	NMS Z.2003.41.1	Figueirido et al. 2021	Occasion al	interm ediate
Ursus maritimus	**Ursidae**	MNCN 21570	Figueirido et al. 2021	Pounce	interm ediate
Arctictis binturong	**Viverridae**	UVA 5474	Figueirido et al. 2021	Occasion al	slow
Genetta genetta	**Viverridae**	UVA 1488	Figueirido et al. 2021	Pounce	interm ediate
Genetta tigrina	**Viverridae**	UVA 3824	Figueirido et al. 2021	Pounce	interm ediate
Paradoxurus hermaphroditus	**Viverridae**	UVA 4610	Figueirido et al. 2021	Occasion al	interm ediate

Suppl. Table 2. Detailed information on the scanned fossil specimen; their museum specimen numbers, source and elements.

**Taxa**	**Family**	**Element**	**ID**	**Source**
Aenocyon dirus	Canidae	CF	ROM 27396	Scanned by JAS at the Royal Ontario Museum, Toronto
		CM	ROM 27460	
		CL	ROM 27484	
		TL	ROM 27614	
		LF	ROM 27659	
		LM	ROM 27734	

2. Principal component analysis

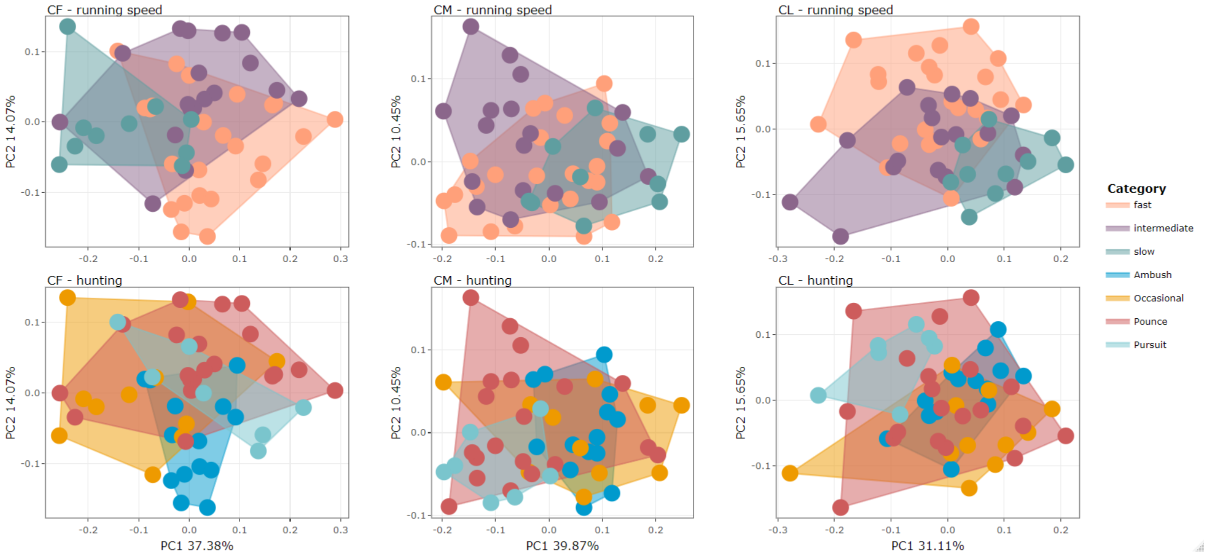

Suppl. Figure 1. Principal component analysis for single cervical vertebrae (CF, CM, CL) with running speed and hunting behaviour morphospaces. For details on the specimens see interactive plot:

https://juliaaschwab.github.io/Vertebrae_locomotion_plots/PCA_cervicals_individual.html

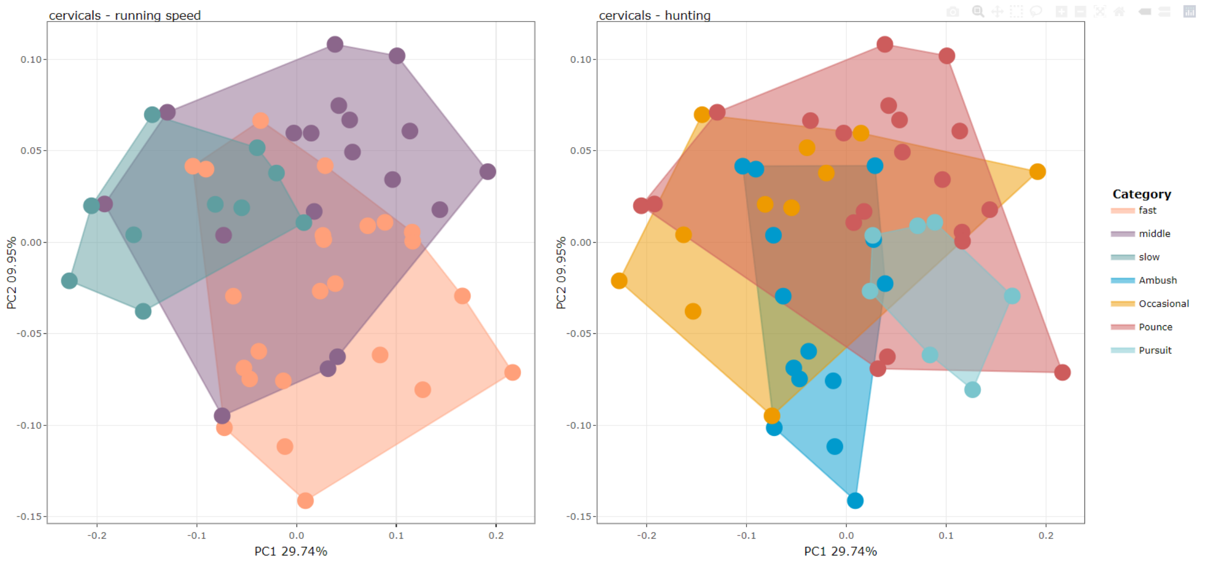

Suppl. Figure 2. Principal component analysis for combined cervical vertebrae with running speed and hunting behaviour morphospaces. For details on the specimens see interactive plot:

https://juliaaschwab.github.io/Vertebrae_locomotion_plots/PCA_cervicals_all.html

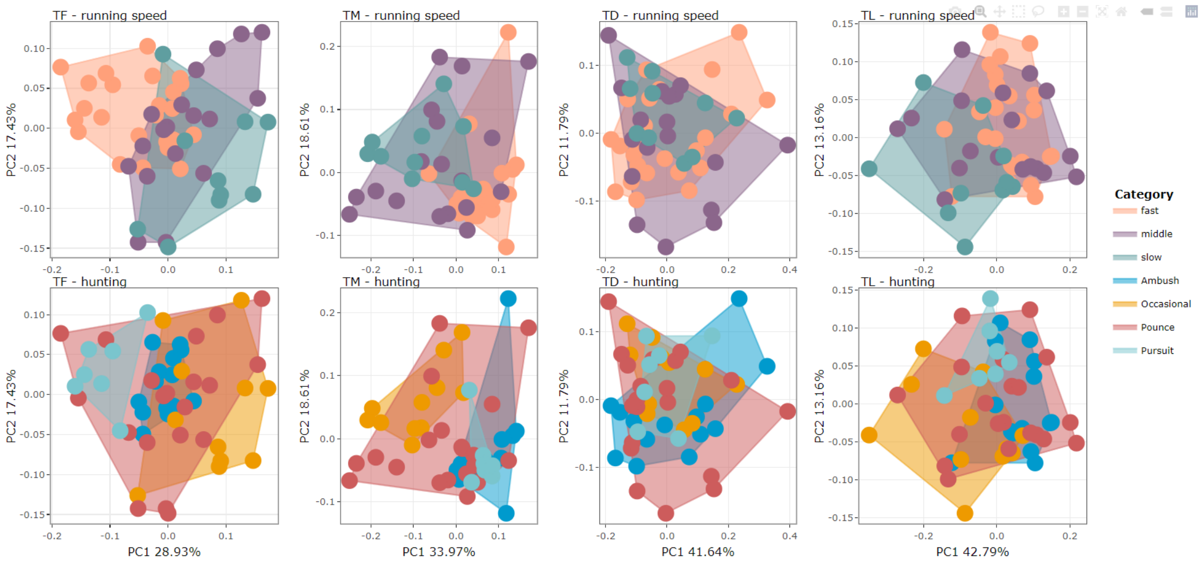

Suppl. Figure 3. Principal component analysis for single thoracic vertebrae (TF, TM, TD, TL) with running speed and hunting behaviour morphospaces. For details on the specimens see interactive plot:

https://juliaaschwab.github.io/Vertebrae_locomotion_plots/PCA_thoracics_individual.html

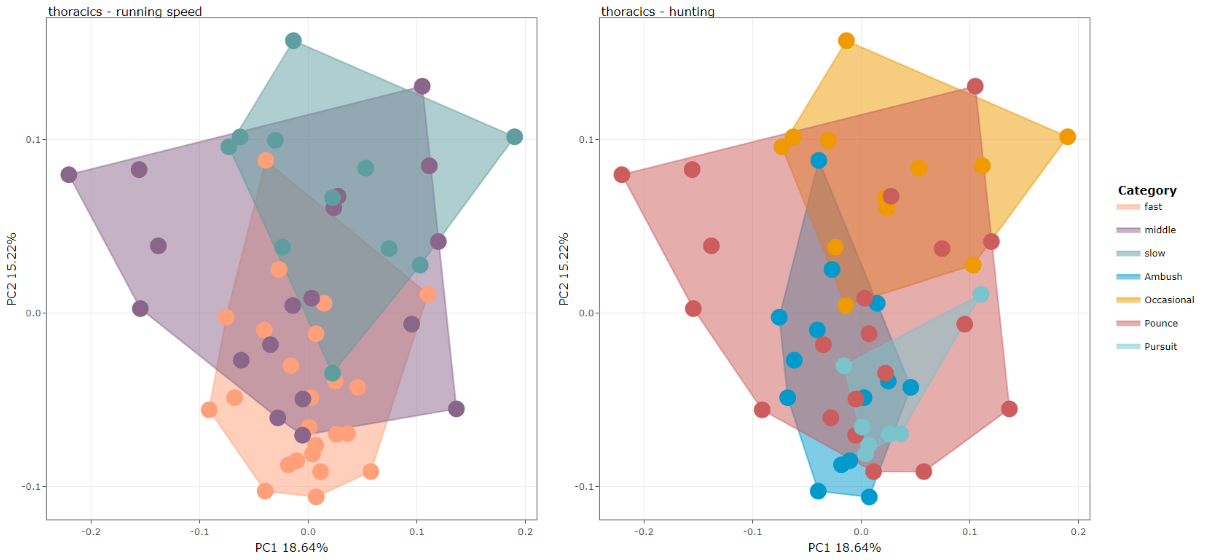

Suppl. Figure 4. Principal component analysis for combined thoracic vertebrae with running speed and hunting behaviour morphospaces. For details on the specimens see interactive plot:

https://juliaaschwab.github.io/Vertebrae_locomotion_plots/PCA_thoracics_all.html

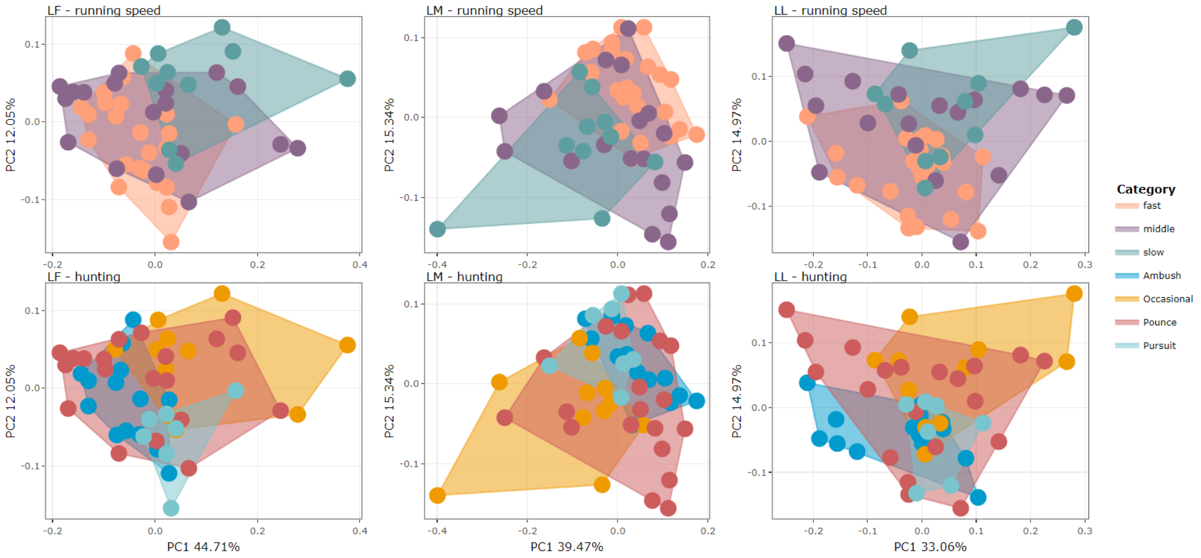

Suppl. Figure 5. Principal component analysis for single lumbar vertebrae (LF, LM, LL) with running speed and hunting behaviour morphospaces. For details on the specimens see interactive plot:

https://juliaaschwab.github.io/Vertebrae_locomotion_plots/PCA_lumbars_individual.html

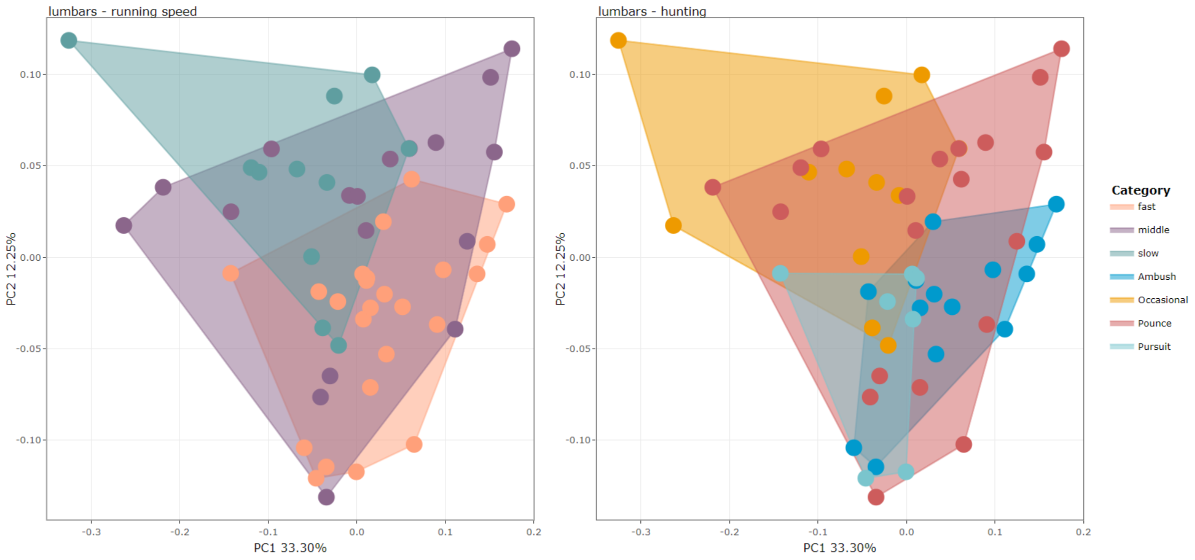

Suppl. Figure 6. Principal component analysis for combined lumbar vertebrae with running speed and hunting behaviour morphospaces. For details on the specimens see interactive plot:

https://juliaaschwab.github.io/Vertebrae_locomotion_plots/PCA_lumbars_all.html

4. Morphospace clustering analysis

Suppl. Table 3. Results of the PERMANOVA, for the cervical region, to test which running speeds/hunting behaviours are statistically different. Highlighted in bold are p-value ≤ 0.005.

Region	Ecology	pair	R2	p value	F
**cervical**	Hunting	Ambush vs Pounce	0.139016	**0.001**	5.166771
**cervical**	Hunting	Occasional vs Ambush	0.127488	0.011	3.360668
**cervical**	Hunting	Occasional vs Pounce	0.113534	0.02	3.714179
**cervical**	Hunting	Pursuit vs Ambush	0.310022	**0.001**	8.53709
**cervical**	Hunting	Pursuit vs Occasional	0.275794	0.008	6.093147
**cervical**	Hunting	Pursuit vs Pounce	0.086615	0.066	2.370725
**cervical**	Running	fast vs intermediate	0.07824	0.009	3.395254
**cervical**	Running	fast vs slow	0.227979	**0.001**	9.449665
**cervical**	Running	slow vs intermediate	0.208826	0.007	6.862553
**CF**	Hunting	Ambush vs Pounce	0.125997	0.007	4.613128
**CF**	Hunting	Occasional vs Ambush	0.170926	**0.005**	4.741785
**CF**	Hunting	Occasional vs Pounce	0.123496	0.022	4.086001
**CF**	Hunting	Pursuit vs Ambush	0.145692	0.011	3.240211
**CF**	Hunting	Pursuit vs Occasional	0.179903	0.023	3.509888
**CF**	Hunting	Pursuit vs Pounce	0.050552	0.241	1.331092
**CF**	Running	fast vs intermediate	0.065112	0.034	2.785865
**CF**	Running	fast vs slow	0.225585	**0.001**	9.321511
**CF**	Running	slow vs intermediate	0.219024	**0.001**	7.291666
**CM**	Hunting	Ambush vs Pounce	0.143972	**0.003**	5.381971
**CM**	Hunting	Occasional vs Ambush	0.0351	0.488	0.836665
**CM**	Hunting	Occasional vs Pounce	0.105914	0.016	3.435341
**CM**	Hunting	Pursuit vs Ambush	0.395244	**0.001**	12.41764
**CM**	Hunting	Pursuit vs Occasional	0.253499	0.006	5.433338
**CM**	Hunting	Pursuit vs Pounce	0.063923	0.142	1.707192
**CM**	Running	fast vs intermediate	0.048327	0.088	2.031233
**CM**	Running	fast vs slow	0.132043	0.008	4.868188
**CM**	Running	slow vs intermediate	0.166148	0.009	5.18059
**CL**	Hunting	Ambush vs Pounce	0.082345	0.026	2.871498
**CL**	Hunting	Occasional vs Ambush	0.131579	**0.005**	3.484854
**CL**	Hunting	Occasional vs Pounce	0.060313	0.127	1.861339
**CL**	Hunting	Pursuit vs Ambush	0.263981	**0.001**	6.814564
**CL**	Hunting	Pursuit vs Occasional	0.275476	**0.002**	6.083479
**CL**	Hunting	Pursuit vs Pounce	0.112608	0.022	3.172443
**CL**	Running	fast vs intermediate	0.083868	0.009	3.661816
**CL**	Running	fast vs slow	0.226501	**0.001**	9.370442

**CL**	Running	slow vs intermediate	0.15243	**0.002**	4.675918

Suppl. Table 4. Results of the PERMANOVA, for thoracic vertebrae, to test which running speeds/hunting behaviours are statistically different. Highlighted in bold are p-value ≤ 0.005.

Region	Ecology	pair	R2	p value	F
**thoracic**	Hunting	Ambush vs Pounce	0.078255	0.029	2.716768
**thoracic**	Hunting	Occasional vs Ambush	0.278319	**0.001**	9.255673
**thoracic**	Hunting	Occasional vs Pounce	0.100979	0.026	3.369615
**thoracic**	Hunting	Pursuit vs Ambush	0.171996	0.014	3.946736
**thoracic**	Hunting	Pursuit vs Occasional	0.368755	**0.001**	9.930904
**thoracic**	Hunting	Pursuit vs Pounce	0.105533	0.018	2.949614
**thoracic**	Running	fast vs intermediate	0.088541	**0.004**	3.885702
**thoracic**	Running	fast vs slow	0.244532	**0.001**	10.68151
**thoracic**	Running	slow vs intermediate	0.057123	0.167	1.635766
**TF**	Hunting	Ambush vs Pounce	0.060577	0.085	2.063468
**TF**	Hunting	Occasional vs Ambush	0.221011	**0.001**	6.525452
**TF**	Hunting	Occasional vs Pounce	0.077747	0.068	2.444736
**TF**	Hunting	Pursuit vs Ambush	0.328981	**0.001**	9.315164
**TF**	Hunting	Pursuit vs Occasional	0.436698	**0.001**	12.40395
**TF**	Hunting	Pursuit vs Pounce	0.154511	**0.002**	4.568688
**TF**	Running	fast vs intermediate	0.127879	**0.001**	5.865196
**TF**	Running	fast vs slow	0.257677	**0.001**	11.10791
**TF**	Running	slow vs intermediate	0.059245	0.188	1.637369
**TM**	Hunting	Ambush vs Pounce	0.1186	**0.002**	4.305883
**TM**	Hunting	Occasional vs Ambush	0.354301	**0.001**	12.62032
**TM**	Hunting	Occasional vs Pounce	0.114479	0.019	3.749096
**TM**	Hunting	Pursuit vs Ambush	0.046211	0.483	0.920544
**TM**	Hunting	Pursuit vs Occasional	0.414542	**0.001**	11.32901
**TM**	Hunting	Pursuit vs Pounce	0.116093	0.032	3.283521
**TM**	Running	fast vs intermediate	0.143776	**0.001**	6.716743
**TM**	Running	fast vs slow	0.283849	**0.001**	12.68329
**TM**	Running	slow vs intermediate	0.023128	0.63	0.615569
**TD**	Hunting	Ambush vs Pounce	0.022204	0.527	0.726648
**TD**	Hunting	Occasional vs Ambush	0.065129	0.171	1.602322
**TD**	Hunting	Occasional vs Pounce	0.045349	0.239	1.377578
**TD**	Hunting	Pursuit vs Ambush	0.075913	0.186	1.560827
**TD**	Hunting	Pursuit vs Occasional	0.164102	0.027	3.1411
**TD**	Hunting	Pursuit vs Pounce	0.032277	0.477	0.833834
**TD**	Running	fast vs intermediate	0.016538	0.559	0.672662
**TD**	Running	fast vs slow	0.058016	0.143	1.970849
**TD**	Running	slow vs intermediate	0.030044	0.488	0.805329
**TL**	Hunting	Ambush vs Pounce	0.05048	0.147	1.701221

**TL**	Hunting	Occasional vs Ambush	0.226724	**0.005**	6.743596
**TL**	Hunting	Occasional vs Pounce	0.100982	0.042	3.257409
**TL**	Hunting	Pursuit vs Ambush	0.193976	**0.003**	4.572488
**TL**	Hunting	Pursuit vs Occasional	0.176836	0.017	3.437201
**TL**	Hunting	Pursuit vs Pounce	0.081578	0.1	2.220606
**TL**	Running	fast vs intermediate	0.028804	0.29	1.186322
**TL**	Running	fast vs slow	0.191481	**0.002**	7.578515
**TL**	Running	slow vs intermediate	0.091986	0.09	2.633932

Suppl. Table 5. Results of the PERMANOVA, for lumbar vertebrae, to test which running speeds/hunting behaviours are statistically different. Highlighted in bold are p-value ≤ 0.005.

**Region**	**Ecology**	**pair**	**R2**	**p value**	**F**
**lumbar**	Hunting	Ambush vs Pounce	0.047002	0.167	1.578237
**lumbar**	Hunting	Occasional vs Ambush	0.278492	**0.001**	9.263671
**lumbar**	Hunting	Occasional vs Pounce	0.090731	0.031	2.993546
**lumbar**	Hunting	Pursuit vs Ambush	0.132869	0.051	2.911332
**lumbar**	Hunting	Pursuit vs Occasional	0.159801	0.015	3.233303
**lumbar**	Hunting	Pursuit vs Pounce	0.067226	0.123	1.801773
**lumbar**	Running	fast vs intermediate	0.07065	0.029	3.040822
**lumbar**	Running	fast vs slow	0.231331	**0.001**	9.931331
**lumbar**	Running	slow vs intermediate	0.056503	0.16	1.616941
**LF**	Hunting	Ambush vs Pounce	0.04803	0.18	1.614505
**LF**	Hunting	Occasional vs Ambush	0.261381	**0.002**	8.139177
**LF**	Hunting	Occasional vs Pounce	0.100427	0.051	3.237515
**LF**	Hunting	Pursuit vs Ambush	0.232573	**0.001**	5.758065
**LF**	Hunting	Pursuit vs Occasional	0.169818	0.017	3.272871
**LF**	Hunting	Pursuit vs Pounce	0.101278	0.055	2.817278
**LF**	Running	fast vs intermediate	0.034694	0.215	1.437644
**LF**	Running	fast vs slow	0.230776	**0.001**	9.600363
**LF**	Running	slow vs intermediate	0.091635	0.076	2.622871
**LM**	Hunting	Ambush vs Pounce	0.057256	0.1	1.943479
**LM**	Hunting	Occasional vs Ambush	0.243805	**0.001**	7.415428
**LM**	Hunting	Occasional vs Pounce	0.125782	0.016	4.172502
**LM**	Hunting	Pursuit vs Ambush	0.092067	0.111	1.926652
**LM**	Hunting	Pursuit vs Occasional	0.141857	0.045	2.644903
**LM**	Hunting	Pursuit vs Pounce	0.092546	0.059	2.549598
**LM**	Running	fast vs intermediate	0.076321	0.037	3.305099
**LM**	Running	fast vs slow	0.197623	**0.001**	7.881526
**LM**	Running	slow vs intermediate	0.06884	0.14	1.922156
**LL**	Hunting	Ambush vs Pounce	0.052643	0.122	1.778202
**LL**	Hunting	Occasional vs Ambush	0.206071	**0.002**	5.969839
**LL**	Hunting	Occasional vs Pounce	0.039429	0.291	1.190386

**LL**	Hunting	Pursuit vs Ambush	0.074327	0.198	1.525608
**LL**	Hunting	Pursuit vs Occasional	0.112988	0.109	2.038097
**LL**	Hunting	Pursuit vs Pounce	0.029111	0.587	0.749594
**LL**	Running	fast vs intermediate	0.072948	0.026	3.147505
**LL**	Running	fast vs slow	0.140687	**0.001**	5.239065
**LL**	Running	slow vs intermediate	0.017023	0.76	0.450276

Suppl. Table 6. Results of the PERMANOVA, for the full column, to test which running speeds/hunting behaviours are statistically different. Highlighted in bold are p-value ≤ 0.005.

**Region**	**Ecology**	**pair**	**R2**	**p value**	**F**
**all**	Hunting	Pursuit vs Occasional	0.303078	**0.001**	6.958079
**all**	Hunting	Pursuit vs Ambush	0.246049	**0.001**	6.200567
**all**	Hunting	Occasional vs Ambush	0.271727	**0.001**	8.581563
**all**	Running	fast vs slow	0.26945	**0.001**	11.80261
**all**	Running	fast vs intermediate	0.095217	**0.003**	4.209475
**all**	Hunting	Ambush vs Pounce	0.10963	**0.004**	3.940113
**all**	Hunting	Occasional vs Pounce	0.115309	0.013	3.779805
**all**	Running	slow vs intermediate	0.116364	0.02	3.423889
**all**	Hunting	Pursuit vs Pounce	0.09192	0.048	2.530618

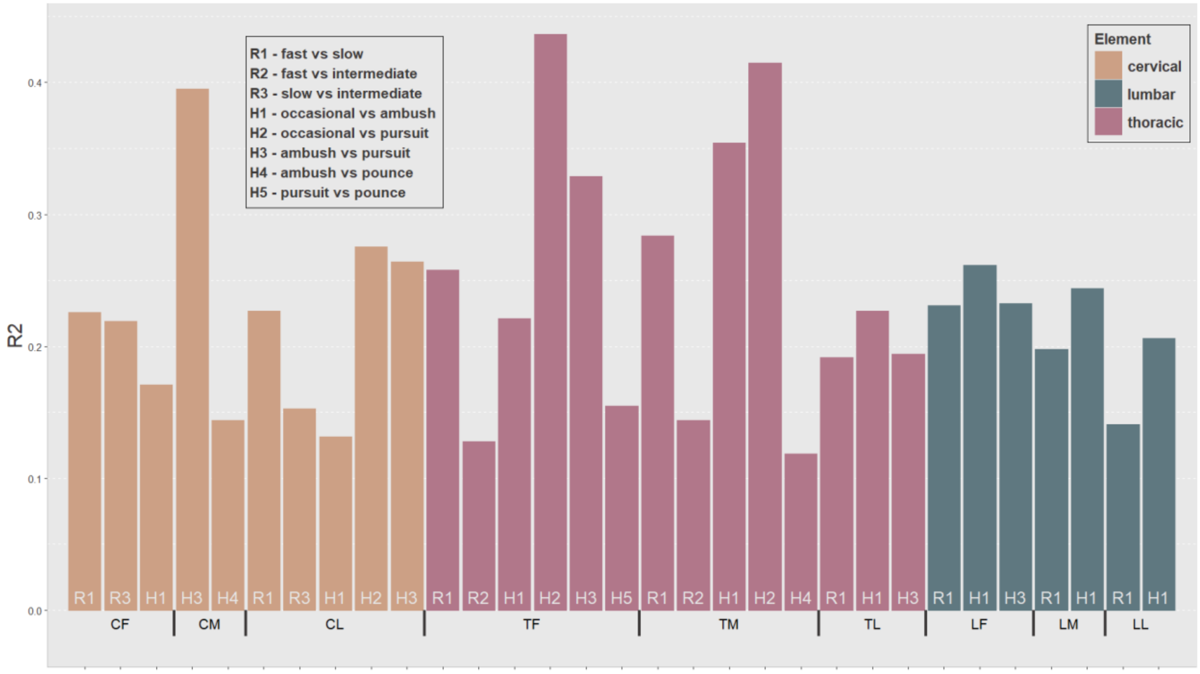

Suppl. Figure 7. PERMANOVA for individual vertebrae. Only significant (p-value ≤ 0.005) results are shown.

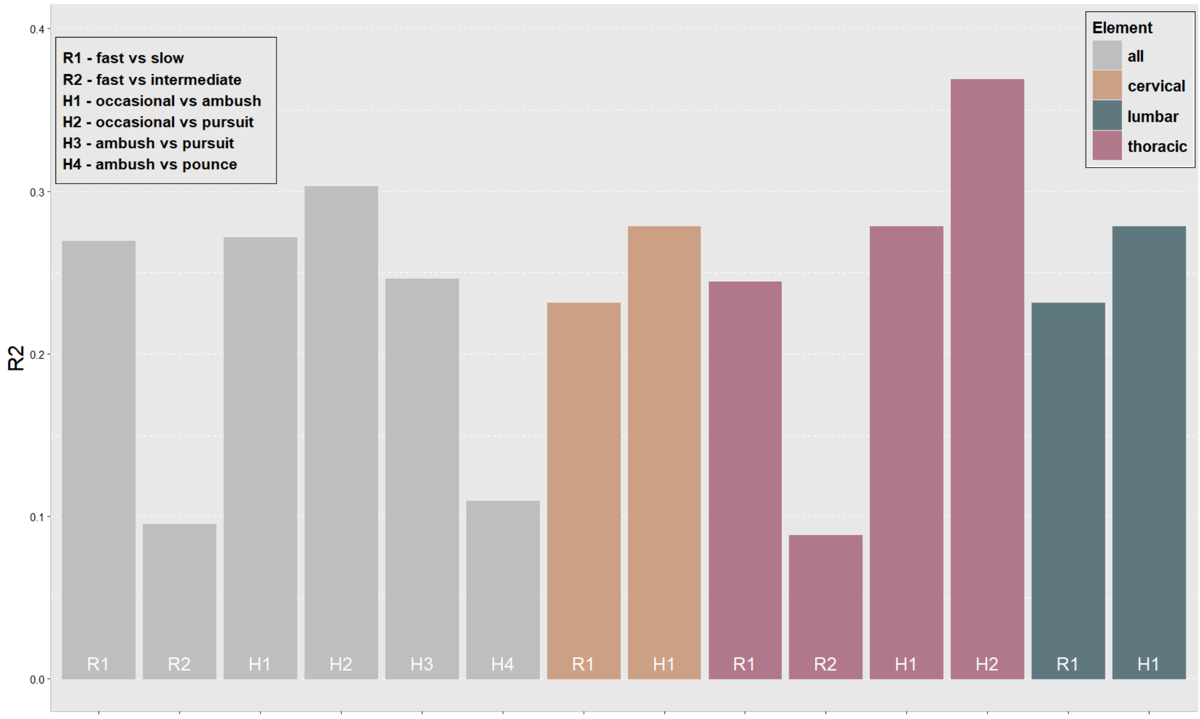

Suppl. Figure 8. PERMANOVA for combined vertebrae. Only significant (p-value ≤ 0.005) results are shown.

5. pGLS regressions

Suppl. Table 7. Results of the PGLS, for cervical vertebrae, to test for correlation which running speeds, hunting behaviours and centroid size. Highlighted in bold are p-value ≤ 0.005 and the highest R^2.

Region	pair	R^2	p-value
**CF**	Csize	0.07643	0.02
**CF**	hunting	0.13738	0.024
**CF**	running	0.17483	**0.001**
**CM**	Csize	0.07907	0.018
**CM**	hunting	0.1261	0.053
**CM**	running	0.17224	**0.001**
**CL**	Csize	0.08256	0.015
**CL**	hunting	0.12341	0.069
**CL**	running	0.16167	**0.001**
**cervical**	hunting	0.11226	0.088
**cervical**	running	0.13475	**0.004**

Suppl. Table 8. Results of the PGLS, for thoracic vertebrae, to test for correlation which running speeds, hunting behaviours and centroid size. Highlighted in bold are p-value ≤ 0.005 and the highest R^2.

Region	pair	R^2	p-value
**TF**	Csize	0.07688	0.027
**TF**	hunting	0.14391	0.028
**TF**	running	0.17998	**0.001**
**TM**	Csize	0.08063	0.019
**TM**	hunting	0.13601	0.039
**TM**	running	0.15574	0.006
**TD**	Csize	0.06481	0.044
**TD**	hunting	0.12573	0.056
**TD**	running	0.1348	0.008
**TL**	Csize	0.10461	0.009
**TL**	hunting	0.1527	0.016
**TL**	running	0.1718	**0.002**
**thoracic**	hunting	0.13908	0.021
**thoracic**	running	0.16697	**0.001**

Suppl. Table 9. Results of the PGLS, for lumbar vertebrae, to test for correlation which running speeds, hunting behaviours and centroid size. Highlighted in bold are p-value ≤ 0.005 and the highest R^2.

Region	pair	R^2	p-value
**LF**	Csize	0.10996	0.009
**LF**	hunting	0.17163	0.005
**LF**	running	0.18183	**0.001**
**LM**	Csize	0.10998	0.011
**LM**	hunting	0.13795	0.036
**LM**	running	0.18004	**0.001**
**LL**	Csize	0.08753	0.013
**LL**	hunting	0.13358	0.024
**LL**	running	0.14934	**0.002**
**lumbar**	hunting	0.12842	0.041
**lumbar**	running	0.1611	**0.001**

Suppl. Table 10. Results of the PGLS, for all combined vertebrae as well as different tested combinations, to test for correlation which running speeds, hunting behaviours and centroid size. Highlighted in bold are p-value ≤ 0.005 and the highest R^2.

Region	pair	R^2	p-value
**all**	hunting	0.11226	0.088
**all**	running	0.13475	**0.004**
**CF, CL**	running	0.13851	**0.004**
**CF, CM**	running	0.1352	**0.005**
**CF, LF**	running	0.14957	**0.001**
**CF, LL**	running	0.14834	**0.001**
**CF, LM**	running	0.1571	**0.001**
**CF, TD**	running	0.14016	**0.001**
**CF, TF**	running	0.15458	**0.001**
**CF, TL**	running	0.14552	**0.001**
**CF, TM**	running	0.16178	**0.001**
**CM, CL**	running	0.13027	**0.005**
**CM, LF**	running	0.14191	**0.004**
**CM, LL**	running	0.14144	**0.001**
**CM, LM**	running	0.15	**0.001**
**CM, TD**	running	0.13231	**0.003**
**CM, TF**	running	0.14689	**0.002**
**CM, TL**	running	0.13785	**0.004**
**CM, TM**	running	0.15504	**0.001**
**CL, LF**	running	0.14555	**0.003**
**CL, LL**	running	0.14468	**0.001**
**CL, LM**	running	0.15366	**0.001**

**CL, TD**	running	0.13571	**0.002**
**CL, TF**	running	0.15074	**0.002**
**CL, TL**	running	0.14135	**0.003**
**CL, TM**	running	0.15869	**0.001**
**TF, LF**	running	0.16346	**0.001**
**TF, LL**	running	0.16049	**0.001**
**TF, LM**	running	0.17182	**0.001**
**TF, TD**	running	0.15214	**0.001**
**TF, TL**	running	0.15843	**0.001**
**TF, TM**	running	0.17693	**0.001**
**TM, LF**	running	0.17065	**0.001**
**TM, LL**	running	0.16708	**0.001**
**TM, LM**	running	0.17837	**0.001**
**TM, TD**	running	0.15972	**0.001**
**TM, TL**	running	0.16573	**0.001**
**TD, LF**	running	0.14708	**0.001**
**TD, LL**	running	0.14608	**0.001**
**TD, LM**	running	0.15488	**0.001**
**TD, TL**	running	0.14298	**0.002**
**TL, LF**	running	0.153	**0.002**
**TL, LL**	running	0.15135	**0.002**
**TL, LM**	running	0.16087	**0.002**
**LF, LL**	running	0.15542	**0.002**
**LF, LM**	running	0.1657	**0.002**
**LM, LL**	running	0.16263	**0.001**
**TF, LF, LM**	running	0.16699	**0.001**
**TF, TL, LF**	running	0.15816	**0.002**
**TF, TL, LM**	running	0.1636	**0.001**
**TF, TM, LF**	running	0.17041	**0.001**
**TF, TM, LM**	running	0.17577	**0.001**
**TM, LF, LM**	running	0.17162	**0.001**
**TL, LF, LM**	running	0.15982	**0.002**
**TF, TL, LF, LM**	running	0.16211	**0.002**
**TF, TM, LF, LM**	running	0.17122	**0.001**

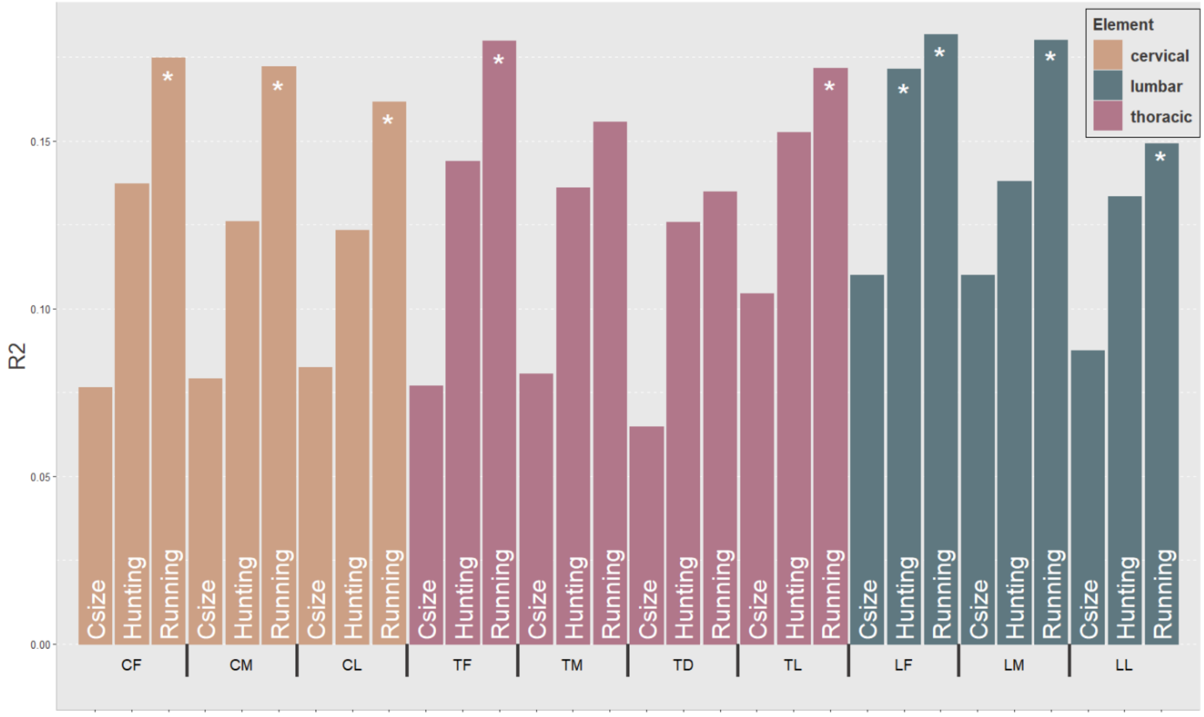

Suppl. Figure 9. PGLS for single vertebrae. Significant (p-value ≤ 0.005) results are shown with an asterisk (*).

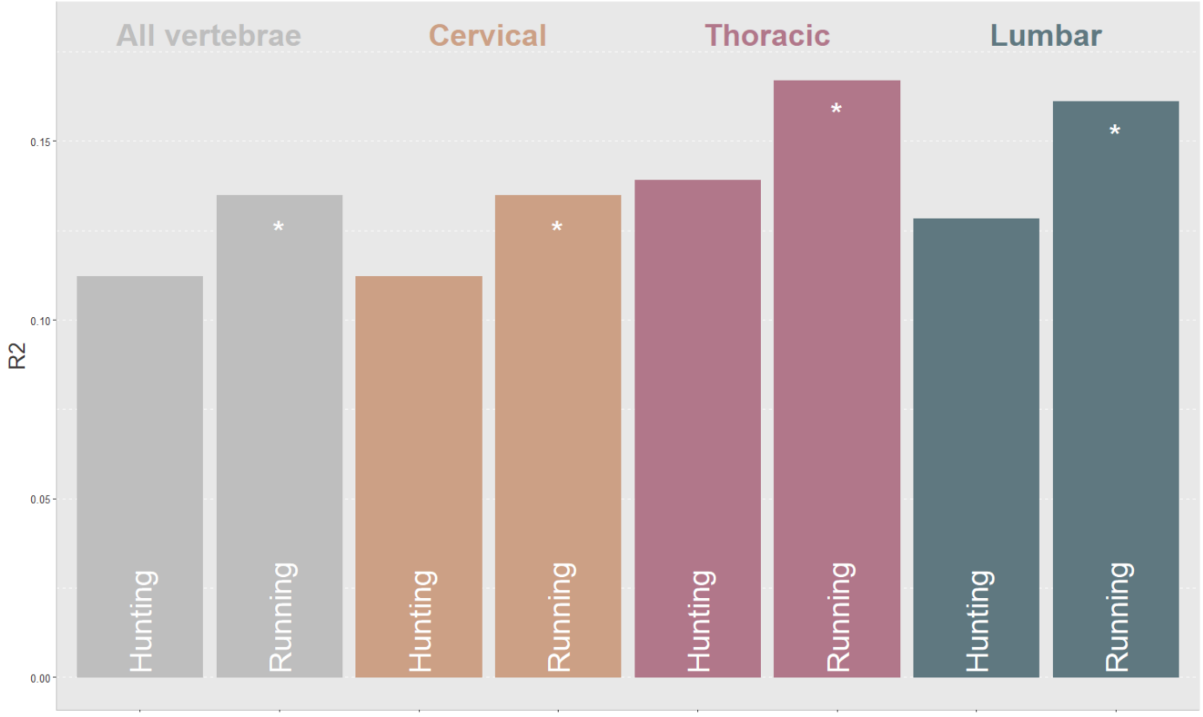

Suppl. Figure 10. PGLS for combined vertebrae. Significant (p-value ≤ 0.005) results are shown with an asterisk (*).

6. Canonical variate analysis

Suppl. Table 11. Overall classification accuracy of different vertebrae for running speed based on a canonical variate analysis (CVA).

CF	fast	intermediate	slow	overall classification accuracy: 76.74419 %			
fast	89.4737	5.2632	5.2632				
intermediate	33.3333	66.6667	0				
slow	0	33.3333	66.6667				
CM	fast	intermediate	slow	overall classification accuracy: 53.48837 %			
fast	57.8947	36.8421	5.2632				
intermediate	26.6667	46.6667	26.6667				
slow	11.1111	33.3333	55.5556				
CL	fast	intermediate	slow	overall classification accuracy: 53.48837 %			
fast	78.95	15.79	5.26				
intermediate	33.33	26.67	40.00				
slow	11.11	44.44	44.44				
TL	fast	intermediate	slow	overall classification accuracy: 55.81395 %			
fast	73.684	10.526	15.789				
intermediate	13.333	53.333	33.333				
slow	22.222	55.556	22.222				
LF	fast	intermediate	slow	overall classification accuracy: 60.46512 %			
fast	68.4211	26.3158	5.2632				
intermediate	26.6667	53.3333	20				
slow	11.1111	33.3333	55.5556				
LM	fast	intermediate	slow				
fast	68.421	31.579	0	overall classification accuracy: 51.16279 %			
intermediate	53.333	26.667	20				
slow	11.111	33.333	55.556				

Suppl. Table 12. Overall classification accuracy of different vertebrae for hunting behaviour based on a canonical variate analysis (CVA).

LF	Ambush	Occasional	Pounce	Pursuit	
**Ambush**	58.33	8.33	16.67	16.67	overall classification accuracy: 37.2093 %
**Occasional**	10.00	50.00	30.00	10.00	
**Pounce**	25.00	43.75	18.75	12.50	
**Pursuit**	40.00	0.00	40.00	20.00	

Suppl. Table 13. Overall classification accuracy of all preserved vertebrae (CF, CM, CL, TL, LF and LM) of *Aenocyon dirus*for running speed based on a canonical variate analysis (CVA).

**All**	**fast**	**intermediate**	**slow**	
**fast**	52.6316	42.1053	5.2632	overall classification accuracy: 34.88372 %
**intermediate**	60.0000	26.6667	13.3333	
**slow**	33.3333	55.5556	11.1111	

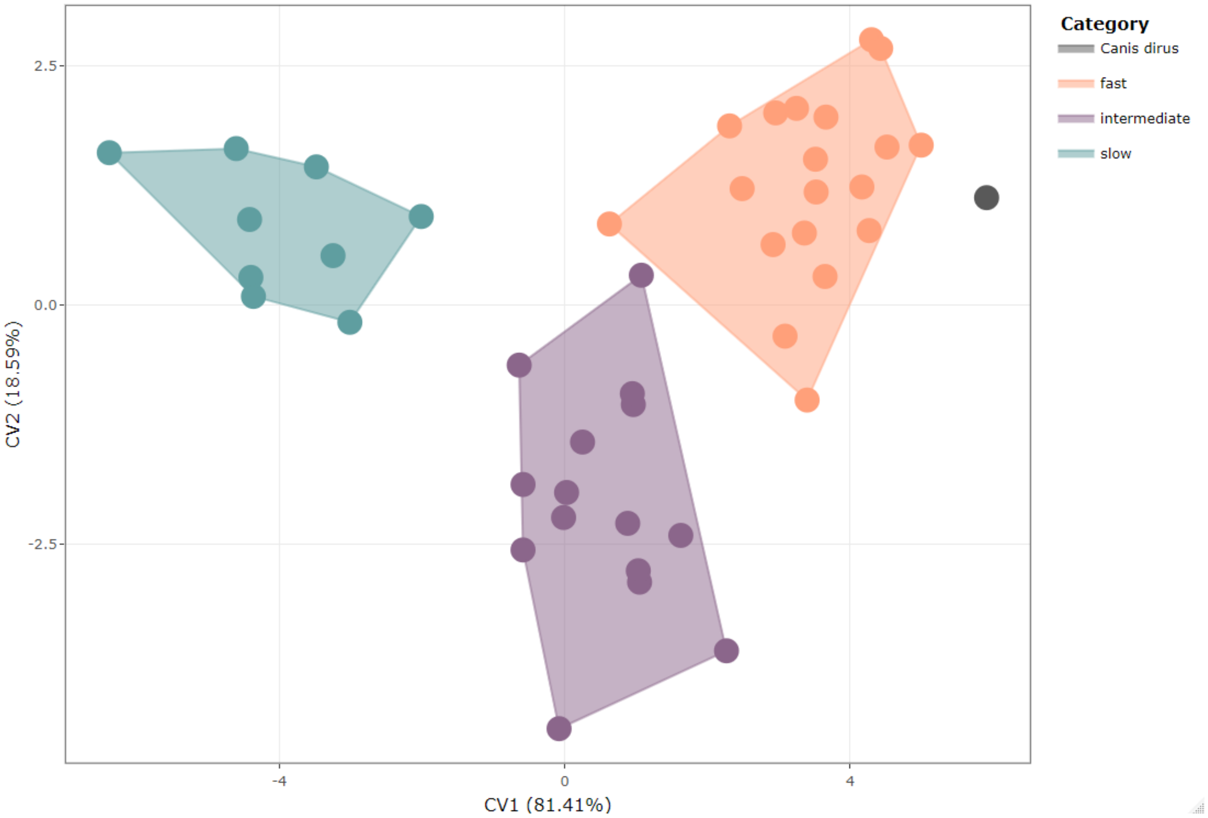

Suppl. Figure 11. CVA of CF and running speed. For details on the specimens see interactive plot:

https://juliaaschwab.github.io/Vertebrae_locomotion_plots/CVA_CF.html

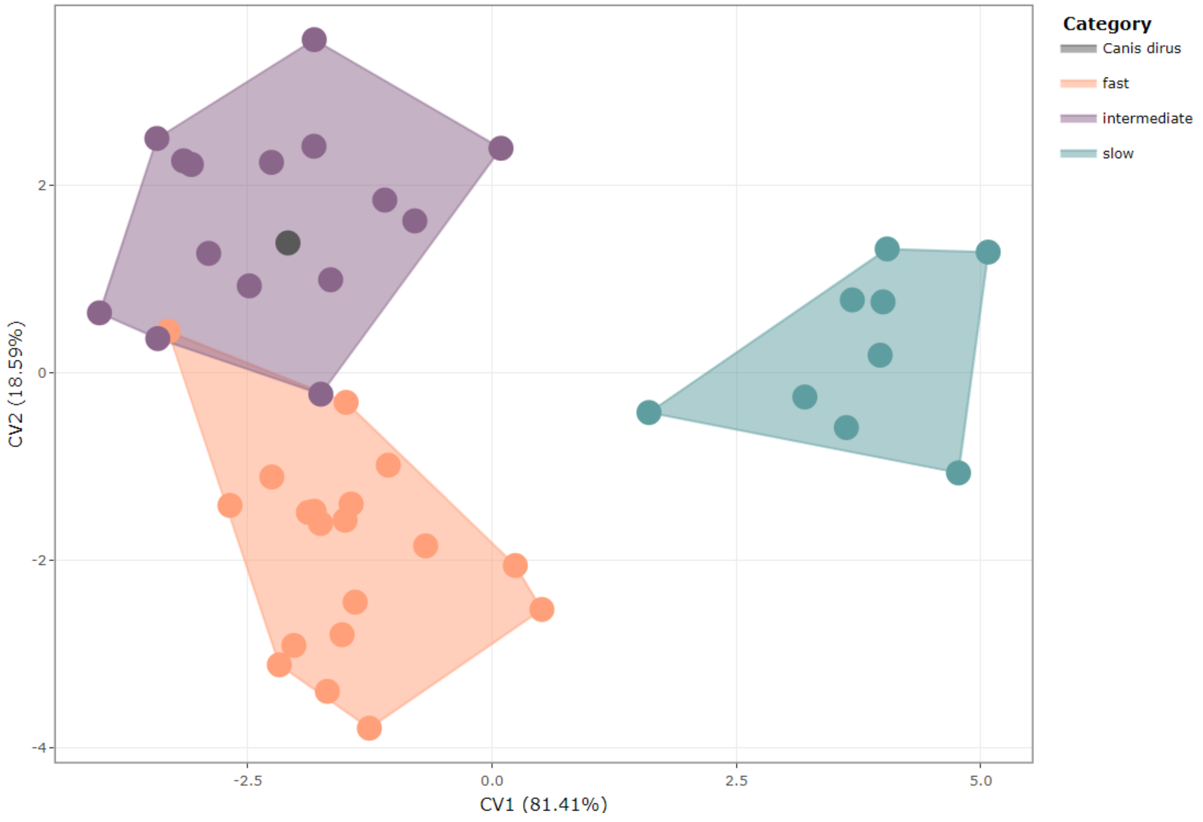

Suppl. Figure 12. CVA of CM and running speed. For details on the specimens see interactive plot:

https://juliaaschwab.github.io/Vertebrae_locomotion_plots/CVA_CM.html

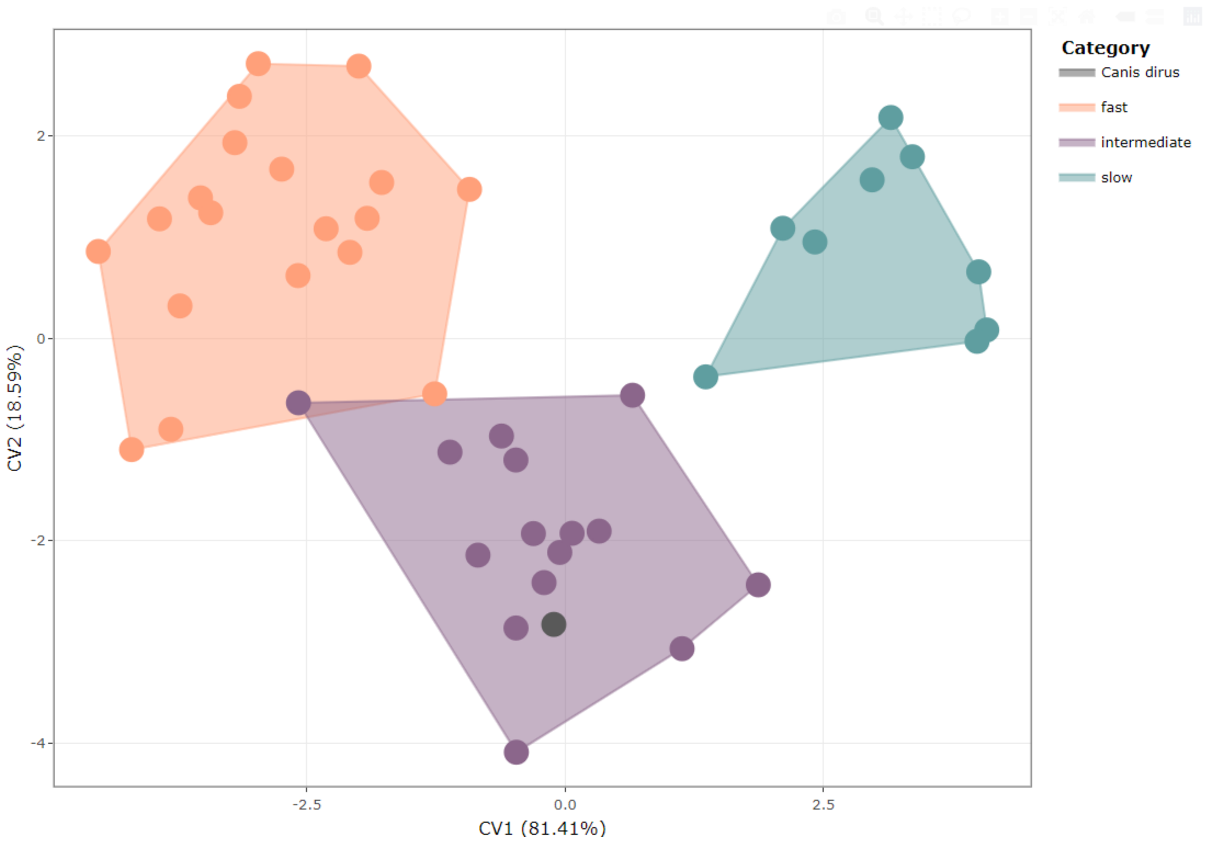

Suppl. Figure 13. CVA of CL and running speed. For details on the specimens see interactive plot:

https://juliaaschwab.github.io/Vertebrae_locomotion_plots/CVA_CL.html

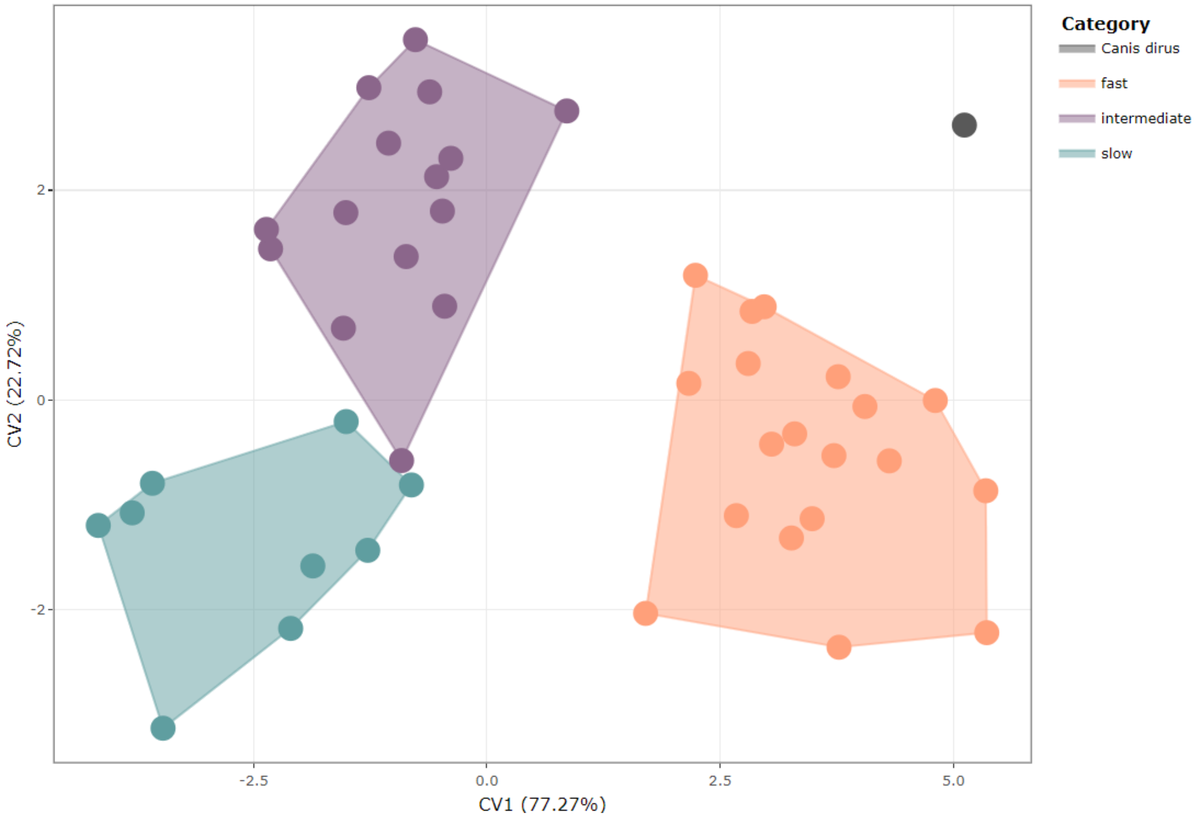

Suppl. Figure 14. CVA of TL and running speed. For details on the specimens see interactive plot:

https://juliaaschwab.github.io/Vertebrae_locomotion_plots/CVA_TL.html

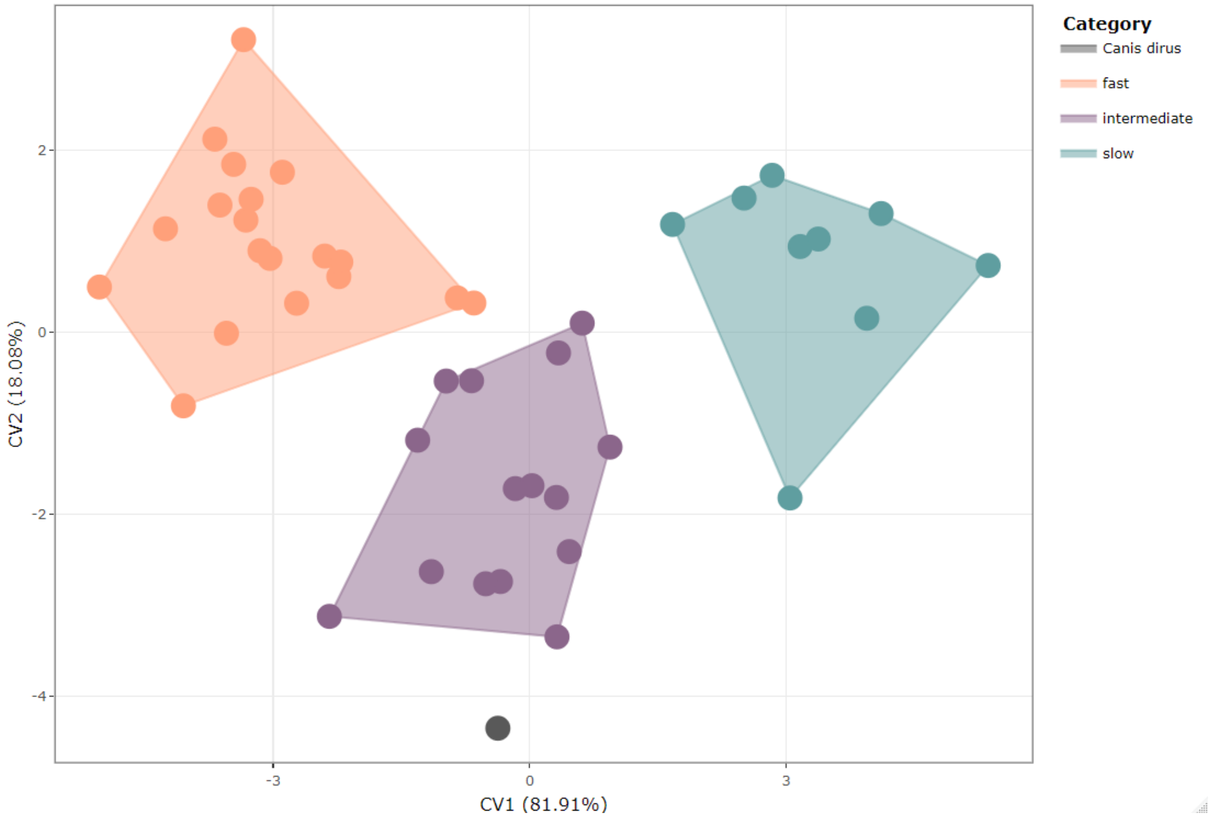

Suppl. Figure 15. CVA of LM and running speed. For details on the specimens see interactive plot:

https://juliaaschwab.github.io/Vertebrae_locomotion_plots/CVA_LM.html

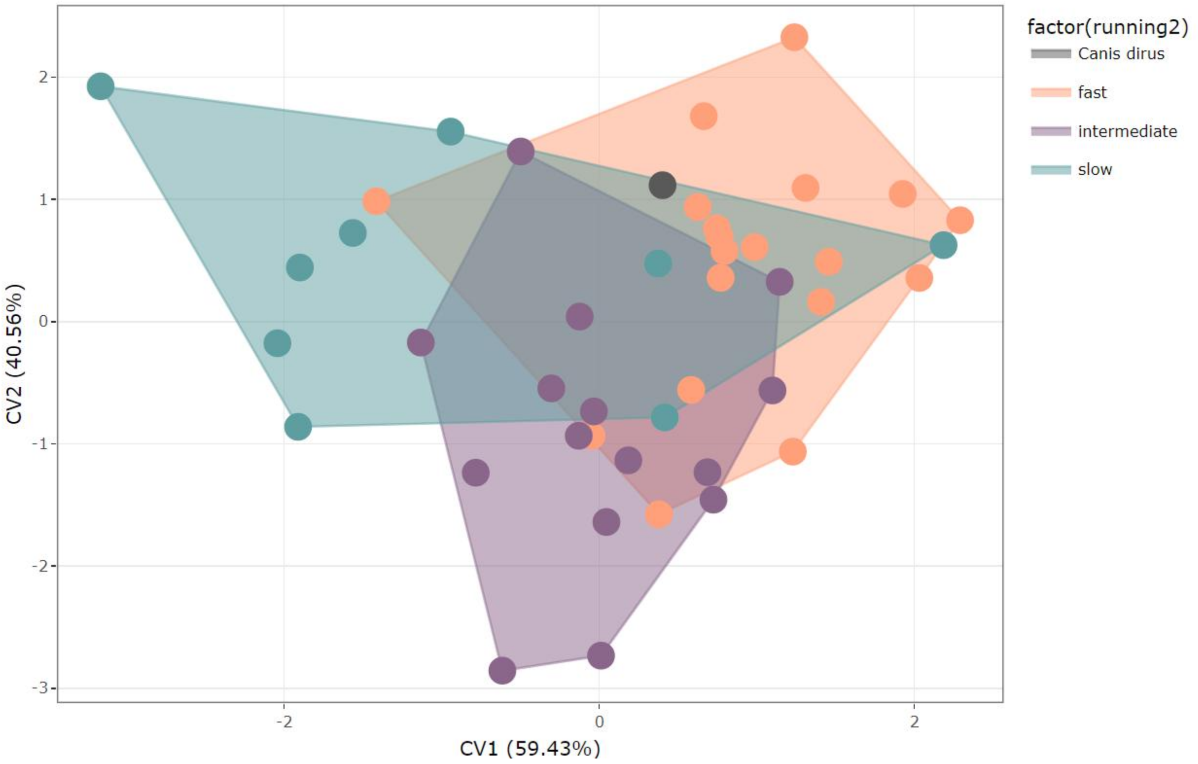

Suppl. Figure 16. CVA of all preserved vertebrae (CF, CM, CL, TL, LF and LM) and running speed. For details on the specimens see interactive plot:

https://juliaaschwab.github.io/Vertebrae_locomotion_plots/CVA_all.html

7. Mahalanobis Distances

Suppl. Table 14. Probability of Mahalanobis Distances for hunting behaviour for *Aenocyon dirus*.

	**Ambush**	**Occasional**	**Pounce**	**Pursuit**
LF	0.0000	0.0000	0.0000	1.0000

Suppl. Table 15. Probability of Mahalanobis Distances for running speed for *Aenocyon dirus*.

	**fast**	**intermediate**	**slow**
CF	1.00000	0.00000	0.00000
CM	0.00711	0.99289	0.00000
CL	0.00012	0.99988	0.00000
TL	1.00000	0.00000	0.00000
LF	0.99903	0.00000	0.00096
LM	0.42353	0.57399	0.00248
all	0.79515	0.09167	0.11318

8. **R Code**

```
#Reading data (using Canis lupus cervicals as an example)

Canis_lupus<-read.morphologika("Landmarks/Cervical/Canis_lupus_UVA 4589_cervical.txt")

#extract First Cervical (CF)

Canis_lupus_CF<-(Canis_lupus[,,c(1)])

#Combine all First Cervicals

CL.raw.data<abind(Acinonyx_jubatus_CF,Ailuropoda_melanoleuca_CF,Ailurus_fulgens_CF,Ar
ctictis_binturong_CF, Canis_lupus_CF,……)


#Procrustes alignment Cervicals

CF.gpagen<-gpagen(CF.raw.data)

 CF.coords<-CF.gpagen$coords


#Morpholocks Code

#organising data into data blocks

CF.block <- formatBlock(CF.coords,k = 3,gpa = F)

CM.block <- formatBlock(CM.coords,k = 3,gpa = F)

CL.block <- formatBlock(CL.coords,k = 3,gpa = F)

TF.block <- formatBlock(TF.coords,k = 3,gpa = F)

TM.block <- formatBlock(TM.coords,k = 3,gpa = F)

TL.block <- formatBlock(TL.coords,k = 3,gpa = F)

TD.block <- formatBlock(TD.coords,k = 3,gpa = F)

LF.block <- formatBlock(LF.coords,k = 3,gpa = F)

LM.block <- formatBlock(LM.coords,k = 3,gpa = F)

LL.block <- formatBlock(LL.coords,k = 3,gpa = F)


#combine blocks into a block list

vertebrae.blocklist<-combineBlocks(blocks = c(CF.block,CM.block,CL.block,
```

```
                              TF.block,TM.block,TL.block,TD.block,
                              LF.block,LM.block,LL.block))

#analysing Blocks and performe principal component analysis on blocklist
vertebrae.result<- analyseBlocks(vertebrae.blocklist, ncomp = 10, option="pca")

#plot PCA
loadingsPlot(vertebrae.result)

#PERMANOVA
PERMANOVA.results.CF<-pairwise.adonis(CF.scores, factors=as.vector(running), sim.method = 'euclidean', p.adjust.m ='fdr')
PERMANOVA.results.CF

#PGLS
CF.gdf <- geomorph.data.frame(CF.gpagen,loco.data)
CF.pgls<- procD.pgls(coords ~ running, phy = tree, data = CF.gdf, iter = 999)
```
